# Comparative analysis through propensity score matching in thyroid cancer: unveiling the impact of multiple malignancies

**DOI:** 10.3389/fendo.2024.1366935

**Published:** 2024-06-04

**Authors:** Akram Al-Ibraheem, Ahmed Saad Abdlkadir, Dhuha Ali Al-Adhami, Egesta Lopci, Amal Al-Omari, Mahmoud Al-Masri, Yacoub Yousef, Nabeela Al-Hajaj, Issa Mohamad, Susanne Singer, Gerasimos P. Sykiotis

**Affiliations:** ^1^ Department of Nuclear Medicine, King Hussein Cancer Center (KHCC), Amman, Jordan; ^2^ Division of Nuclear Medicine, Department of Radiology and Nuclear Medicine, the University of Jordan, Amman, Jordan; ^3^ Nuclear Medicine Unit, IRCCS– Humanitas Clinical and Research Hospital, Rozzano, Milan, Italy; ^4^ Office of Scientific Affairs and Research (OSAR), King Hussein Cancer Center (KHCC), Amman, Jordan; ^5^ Department of Surgery, King Hussein Cancer Center (KHCC), Amman, Jordan; ^6^ Department of Radiation Oncology, King Hussein Cancer Center (KHCC), Amman, Jordan; ^7^ Institute of Medical Biostatistics, Epidemiology and Informatics (IMBEI), University Medical Centre Mainz, Mainz, Germany; ^8^ Department of Endocrinology, Diabetology and Metabolism, Vaud University Hospital Center (CHUV), Lausanne, Switzerland

**Keywords:** thyroid carcinoma, multiple primary neoplasms, survival analysis, prognosis, multiple primary malignancies, multiple primary cancers

## Abstract

**Background:**

The incidence of thyroid cancer is on the rise worldwide, with childhood exposure to radiation being the sole acknowledged catalyst for its emergence. Nonetheless, numerous other factors that may pose risks are awaiting thorough examination and validation. This retrospective study aims to explore the malignancies linked to thyroid cancer and contrast the survival rates of those afflicted with a solitary tumor versus those with multiple primary neoplasms (MPN).

**Methods:**

This retrospective study examined data from King Hussein Cancer Center (KHCC), Jordan. Among 563 patients diagnosed with thyroid cancer, 30 patients had thyroid malignancy as part of MPN. For a 1:3 propensity score-matched analysis, 90 patients with only a primary thyroid malignancy were also enrolled.

**Results:**

Hematologic and breast malignancies were among the most frequent observed cancers alongside thyroid neoplasm. Patients who had MPN were diagnosed at older age, had higher body mass index and presented with higher thyroglobulin antibody levels (*p* < 0.05 for each). Additionally, MPN patient displayed a stronger family history for cancers (*p*= 0.002). A median follow-up duration of 135 months unveiled that MPN patients faced a worse 5-year survival compared to their counterparts with a singular neoplasm (87% vs 100% respectively; p < 0.01). However, no distinction emerged in the 5-year event-free survival between these two groups.

**Conclusion:**

MPN correlates with a significantly altered survival outcome of thyroid cancer patients. The diagnosis of thyroid carcinoma at an older age, accompanied by elevated initial thyroglobulin antibody levels and a notable familial predisposition, may raise concerns about the potential occurrence of synchronous or metachronous tumors.

## 1 Introduction

Thyroid cancer (TC) constitutes 1% of all cancer cases (1–5). The incidence of TC has been increasing in most populations worldwide (6, 7). This increase may be attributed to advancements in clinical practices and/or changes in histological criteria, as well as potential alterations in environmental and hormonal factors (5, 8, 9). While TC is generally more prevalent in females, males have a higher likelihood of having a positive family history of malignancy (10). Treatment options for TC vary depending on the histology and extent of the disease, encompassing surgical intervention, radioactive iodine therapy, suppressive thyroid hormone therapy, and occasionally, external beam radiotherapy (11). Despite acknowledged ethnic variations, the prognosis for treated TC is favorable, with excellent overall survival (OS) rates (5, 12–14).

When considering the histologic subtypes, TC can be divided into five unique classifications: papillary TC (PTC), follicular TC (FTC), medullary TC (MTC), anaplastic TC, and miscellaneous subtypes (15). It is worth noting that the majority of TC, around 95%, originates from the follicular epithelium, manifesting in PTC, FTC, and anaplastic variations (16). In numerous surveyed countries, the most prevalent subtype is PTC, accounting for 70% or more of cases (14, 17, 18). The prognosis is highly favorable for PTC and FTC, whereas other histologic subtypes demonstrate a poorer prognosis and limited therapeutic options (19–22).

As the early cancer detection becomes more common, individuals who survive this disease are living longer, which may increase the risk of developing multiple primary neoplasms (MPN) (23). A limited number of investigations have been conducted to examine the potential risk of developing a second neoplasm following a diagnosis of TC, including comprehensive studies conducted in Europe and the United States (24–28). These studies have consistently demonstrated a rise in the occurrence of cancers subsequent to TC diagnosis. Additionally, it has been observed that TC can be diagnosed as a second primary neoplasm subsequent to other forms of cancer (25, 29–32).

The present study aimed to examine the cancers associated with TC and to compare survival rates between patients with thyroid-only cancers and patients with thyroid cancer as part of MPN. This may help shed light on common etiological factors affecting such a challenging group of patients.

## 2 Materials and methods

### 2.1 Data collection

The medical records of 563 patients diagnosed with TC between September 1998 and December 2021 were retrospectively enrolled. For these patients, demographic data were collected and included age at diagnosis and patient gender. In addition, pathological data included cancer site, cancer type, cancer grade, and cancer stage according to the American Joint Committee on Cancer 8th Edition (AJCC 8th) (33). Moreover, the baseline postoperative levels for serum thyroglobulin and thyroglobulin antibodies were collected (34). Other important details include a history of MPN.

The diagnosis of MPN relies on the criteria put forth by Warren et al. in 1933 (35), which require histopathological confirmation. Furthermore, each individual cancer included in MPN must be identifiable as separate from the surrounding normal mucosa (35). Last, the possibility of the second neoplasm being metastatic should be excluded (35). For those patients, another round of pathological analysis was performed for other cancer subtypes in addition to ranking each and every subtype along with labeling the date of diagnosis for each cancer. According to the criteria set forth by the International Association of Cancer Registries and the International Agency for Research on Cancer (IACR/IARC), synchronous MPN are defined as the diagnosis of two or more primary malignancies within a 6-month timeframe (36). In contrast, metachronous MPN pertain to the presence of more than two primary malignancies emerging beyond the 6-month timeframe (36).

To refine our investigation, the study cohort was further segmented into two distinct groups. The first group consisted of patients diagnosed with MPN who had TC as a component of their MPN (n = 30). A 1:3 propensity score matching strategy was utilized to select a comparable group of patients with only TC. This technique sought to identify individuals with similar characteristics, including gender, chronic diseases (namely diabetes mellitus, hypertension, and dyslipidemia), type of thyroid cancer, and stage. By employing this propensity score matching approach, our objective was to mitigate the influence of any factors that could potentially influence the outcomes. As a result, a cohort of 120 patients was obtained for subsequent analysis.

In light of the absence of a comprehensive genetic analysis within our examined cohort, a meticulous screening process of patient records was undertaken to eliminate subjects exhibiting indications of syndromic manifestations elucidated by Vânia Nosé (37). These manifestations encompass, among others, Multiple Endocrine Neoplasia Type 2, Cowden Syndrome, Familial Adenomatous Polyposis, Carney Complex, and Werner Syndrome (37). Notably, there were no instances of patients presenting with the mentioned syndromes in our studied population.

### 2.2 Ethics consideration

This retrospective study received its approval from the Institutional Review Board of King Hussein Cancer Center (KHCC) in Amman, Jordan (Registration number: 22 KHCC 131, 11 August 2022). Written informed consent for this study was not needed due to its retrospective nature.

### 2.3 Statistical analysis

The statistical analysis was conducted using Stata software version 17 (College Station, TX, USA). To determine the data distribution, a normality test called Shapiro Wilk was utilized. Normally distributed variables were presented as the mean ± standard deviation (SD). The median and interquartile range (IQR) were expressed for variables that did not have a normal distribution. Normally distributed variables are presented as the mean ± standard deviation (SD). The frequencies of categorical variables were represented by percentages (%). To analyze categorical variables, we used either the chi squared test or Fisher exact test while the nonparametric Mann Whitney U test or t-test were used to analyze continuous variables based on their appropriateness. Kaplan−Meier plots and log-rank tests were utilized to illustrate the survival curves. cancer-specific OS and event-free survival (EFS) were calculated. First, we conducted an analysis focused on cancer-specific OS and EFS. In this context, cancer-related OS was defined as the interval from the time of initial diagnosis to the point of death directly attributed to any cancer subtype. Instances of death resulting from unrelated causes were censored. In the same manner, cancer-specific EFS was delineated as the span from the time of diagnosis to the advent of advancing, or recurring cancer. Subsequently, an examination of univariate and multivariate Cox proportional hazard analysis was undertaken to investigate the predictive weight of diverse clinicopathologic factors. A significance threshold of less than 0.05 (*p* < 0.05) was deemed statistically significant.

## 3 Results

This retrospective study utilized KHCC cohort of TC patients to explore patients who had MPN. A total of 30 patients were identified with MPN. Those patients were observed to have 68 neoplasms in total. Thirty-one of these were thyroid neoplasms and the remaining 37 were other malignancies. Demographic and clinicopathologic characteristics are shown in **Table 1**.

**Table 1:** Demographic and histopathologic characteristics for patients with multiple neoplasms.

Demographics	
Age (in Years)	
Median	54 years
Interquartile Range	42–64 years
Gender (Number of Patients, Percentage)	
Male	14, 47%
Female	16, 53%
Thyroid Cancer (Total Neoplasms= 31)	
Histopathologic Subtype (Number of Thyroid Neoplasms, Percentage)	
Papillary	24, 77.3%
Follicular	2, 6.5%
Hurthle	2, 6.5%
Medullary	3, 9.7%
Cancer Stage (Number of Thyroid Neoplasms, Percentage)	
Stage I	20, 64.5
Stage II	10, 32.3%
Stage III	1, 3.2%
Triggers for thyroid cancer discovery (Number of Patients, Percentage)	
Discovered following neck swelling	9, 30%
Discovered incidentally during whole-body imaging	8, 26.7%
Discovered before referral to our institute	4, 13.3%
Diagnosed by an outside institute	4, 13.3%
Incidental discovery on histopathologic examination after surgical removal of ipsilateral thyroid lobe adjacent to the resected tumor of interest	3, 10%
Subclinical hypothyroidism and ultrasound finding of thyroid nodule	2, 6.7%
Prior radiation before thyroid cancer diagnosis (Number of Patients, Percentage)	
Neck Radiotherapy	3, 10%
Diagnostic whole-body Computed tomography	16, 53.3%
Diagnostic whole-body Positron emission tomography/computed tomography	9, 30%
Surgical Intervention (Number of Patients, Percentage)	
Total Thyroidectomy	25, 83.3%
Thyroid lobectomy followed by completion thyroidectomy	3, 10%
Radioactive Iodine (Number, Percentage)	
Number of patients, percentage	27, 90%
Cumulative dose (Median, Interquartile Range)	2.4 GBq (1.1–5.9 GBq)
Doses Received (Mean, Standard Deviation)	1 ± 1
Interval days between total thyroidectomy and radioactive iodine	
Median	29
Interquartile range	17–96
Other Malignancies (Total Neoplasms= 37)	
Classification (Number of Patients, Percentage)	
Synchronous only	10, 33.3%
Metachronous	20, 66.7%
Cancer Stage (Number of Neoplasms, Percentage)	
Stage I	10, 27%
Stage II	11, 29.7%
Stage III	9, 24.3%
Stage IV	7, 19%

### 3.1 Patients with multiple primary neoplasms

#### 3.1.1 Incidence

Between September 1998 and December 2021, of 563 TC patients, 533 patients (95%) had a single thyroid neoplasm. The remaining 30 patients (5%) were diagnosed with MPN. For patients with MPN, a total of 24 patients had two primary neoplasms. In addition, a total of four patients presented with three neoplasms while only two patients had four neoplasms.

#### 3.1.2 Temporal presentation

The median interval between first and second primary neoplasms was 13 months with an IQR of 2–46 months. Of all 30 patients diagnosed with MPN, only one third (n= 10) had synchronous malignancies. The remaining 20 patients had metachronous MPN. TC was the first diagnosed neoplasm in eight patients. However, all remaining patients were diagnosed with TC subsequent to their initial primary neoplasm (**Table 2**).

**Table 2:** Summary table for the identified cancer subtypes in patients with multiple neoplasms.

Coexisting Neoplasm Discovered Concomitantly				
#	First Neoplasm	Coexisting Neoplasms		
**1**	Medullary Thyroid Cancer	Parathyroid Carcinoma		
**2**	Papillary Thyroid Cancer	Hurthle Cell Neoplasm of the Thyroid		
**3**	Papillary Thyroid Cancer	Laryngeal Squamous Carcinoma of Subglottis		
**4**	Papillary Thyroid Cancer	Laryngeal Squamous Carcinoma of Epiglottis		
**5**	Papillary Thyroid Cancer	Malignant Melanoma		
**6**	Invasive Lobular Carcinoma of right Breast	Invasive Cribriform Carcinoma of left Breast		
Other Synchronous Neoplasm				
	First Neoplasm	Second Neoplasm	Interval Between Both neoplasms	
**7**	Hodgkin’s Lymphoma	Hurthle Cell Neoplasm of the Thyroid	1 Month	
**8**	Papillary Thyroid Cancer	Prostate Cancer	1 Month	
**9**	Invasive Lobular Carcinoma of Breast	Papillary Thyroid Cancer	3 Months	
**10**	Nasopharyngeal Carcinoma	Medullary Thyroid Cancer	5 Months	
Patients with Metachronous Malignancies				
#	First Neoplasm	Second Neoplasm	Third Neoplasm	Fourth Neoplasm
**1**	Malignant Ovarian Teratoma	Follicular Thyroid Cancer	Papillary Urothelial Carcinoma	
**2**	Papillary Thyroid Cancer	Invasive Ductal Carcinoma of Breast		
**3**	Cecal Adenocarcinoma	Papillary Thyroid Cancer		
**4**	Papillary Thyroid Cancer	Chordoma		
**5**	Papillary Thyroid Cancer	Invasive Ductal Carcinoma of Breast		
**6**	Invasive Ductal Carcinoma of Breast	Papillary Thyroid Cancer		
**7**	Papillary Thyroid Cancer	Ileal neuroendocrine Tumor		
**8**	Papillary Thyroid Cancer	Lung Adenocarcinoma		
**9**	AML	DLBCL	Papillary Thyroid Cancer	Oral SCC
**10**	Invasive Ductal Carcinoma of Breast	Papillary Thyroid Cancer		
**11**	Papillary Thyroid Cancer	Gastric Adenocarcinoma		
**12**	Hodgkin’s Lymphoma	Papillary Thyroid Cancer	Leiomyosarcoma of Urinary Bladder	
**13**	Jejunal GIST	Papillary Thyroid Cancer		
**14**	Adenocarcinoma of Gallbladder	Papillary Thyroid Cancer		
**15**	Colorectal Cancer	Papillary Thyroid Cancer		
**16**	Follicular Lymphoma	Papillary Thyroid Cancer		
**17**	Maxillary Sinus SCC	Follicular Thyroid Cancer		
**18**	DLBCL	Follicular Lymphoma	Papillary Thyroid Cancer	
**19**	DLBCL	Medullary Thyroid Cancer		
**20**	Papillary Thyroid Cancer	Invasive Ductal Carcinoma of Breast		
Dual-timing malignancy cases (Cases with Metachronous and Synchronous Cancers)				
#	Metachronous neoplasms	Synchronous Neoplasms				
**1**	Basal cell carcinoma of Scalp → First Detected Neoplasm Papillary Thyroid Cancer → Fourth Neoplasm	ILC of right breast coexisting with ICC of left breast				
**2**	Renal Cell Carcinoma of right Kidney → Third neoplasm	Hodgkin’s Lymphoma → First neoplasm Hurtle Cell Neoplasm → Second Neoplasm				

#### 3.1.3 Thyroid cancer subtypes

In all 30 patients presented with MPN, a total of 31 TC neoplasms were observed. Approximately, 24 patients had an established diagnosis of PTC as the predominant histopathologic subtype (n= 24). Follicular and hurtle subtypes were found in 2 patients each. In addition, MTC was diagnosed in 3 patients (**Table 1**). It is noteworthy that one of our MPN patients had an established diagnosis of both synchronous Hurthle and papillary subtypes (**Table 2**).

#### 3.1.4 Coexisting malignancies: by region

A total of 37 coexisting malignancies were observed. Notably, hematologic and breast malignancies were among the most common identifiable coexisting malignancies in patients with MPN (n=8 for each). This was followed by head and neck cancers (n=6). Then an equivalent number of patients who had gastrointestinal and genitourinary malignancies were identified (n= 5 for each). Other cancer subtypes followed in predominance (**Table 3**).

**Table 3:** Region-based summarization of the identified cancer subtypes in patients with multiple neoplasms.

Hematologic Malignancies			
Site	Histopathology	Number (Percentage)	Cancer Stage (Number)
Cervical Lymph node	DLBCL	3 (10%)	II (n=2); IV (n=1)
	AML	1 (3%)	M1 (n=1)
	HL	2 (6.7%)	II (n=1); III (n=1)
	FL	2 (6.7%)	II (n=1); IV (n=1)
Head and Neck Cancers			
Site	Histopathology	Number (Percentage)	Cancer Stage (Number)
Larynx	SCC	2 (6.7%)	III (n=2)
Parathyroid Carcinoma	Parathyroid Carcinoma	1 (3.3%)	II (n=1)
Oral SCC	SCC	1 (3.3%)	IV (n=1)
Maxillary sinus SCC	SCC	1 (3.3%)	III (n=1)
Nasopharynx	Nasopharyngeal Carcinoma	1 (3.3%)	III (n=1)
Breast			
Site	Histopathology	Number (Percentage)	Cancer Stage (Number)
Left Breast	IDC	3 (10%)	I (n=2); II (n=1)
Right Breast	IDC	3 (10%)	II (n=3)
Right Breast	ILC	1 (3.3%)	I (n=1)
Left Breast	ICC	1 (3.3%)	I (n=1)
Gastrointestinal Malignancies			
Site	Histopathology	Number (Percentage)	Cancer Stage (Number)
Colorectal	Adenocarcinoma	1 (3.3%)	II (n=1)
Cecal	Adenocarcinoma	1 (3.3%)	III (n=1)
Ileum	NET	1 (3.3%)	III (n=1)
Jejunum	GIST	1 (3.3%)	IV (n=1)
Stomach	Adenocarcinoma	1 (3.3%)	IV (n=1)
Genitourinary Malignancies			
Site	Histopathology	Number (Percentage)	Cancer Stage (Number)
Left Ovary	Immature malignant Teratoma	1 (3.3%)	I (n=1)
Urinary Bladder	Papillary Urothelial Carcinoma	1 (3.3%)	III (n=1)
Urinary Bladder	Leiomyosarcoma	1 (3.3%)	II (n=1)
Prostate	Adenocarcinoma	1 (3.3%)	IV (n=1)
Kidney	RCC	1 (3.3%)	I (n=1)
Other Cancers			
Site	Histopathology	Number (Percentage)	Cancer Stage (Number)
Left Lower Lung Lobe	Adenocarcinoma	1 (3.3%)	I (n=1)
Skin (Right Inguinal Area)	Melanoma	1 (3.3%)	III (n=1)
Skin (Right Medial Canthus)	Basal Cell Carcinoma	1 (3.3%)	I (n=1)
T3 Vertebra	Chordoma	1 (3.3%)	IV (n=1)
Gallbladder	Adenocarcinoma	1 (3.3%)	I (n=1)

### 3.2 Propensity score matching

As stated before, 1:3 propensity score matching was performed to observe differences in clinical factors and survival outcomes between the two matched subgroups.

#### 3.2.1 Differences between patients with single and multiple neoplasms

When comparing single thyroid neoplasm with MPN, patients who had TC as part of MPN were substantially older; the median age at diagnosis was 54 years vs 41 years for patients with single TC diagnosis (*p*= 0.04). They also had a higher body mass index (*p*= 0.003). In addition, they had a higher baseline thyroglobulin antibody (median value for MPN was 45.5 vs 11 for patients with single TC; *p*= 0.0003). Finally, a significantly higher percentage of patients had a positive family history of malignancy in patients diagnosed with MPN vs patients with a single TC (50% vs 20%; p= 0.002). Notably, the percentage of smokers, recurrence rates, mortality from TC, elapsed duration since postoperative thyroid markers conduction, and elapsed duration since initial TC diagnosis did not differ significantly when comparing both subgroups (**Table 4**).

**Table 4:** Clinicopathologic characteristics for patients with multiple neoplasms compared to those who had single thyroid malignancy.

Variable	Multiple Neoplasm	Single Neoplasm	*p*-value
Age at diagnosis (Years)	54 (42–64)	41 (24–60)	0.04
BMI (kg/m^2^)	27.1 (24.6–29.3)	21 (20.5–23.6)	0.003
Smoking	11 (36.7%)	47 (51.1%)	0.21
Family History	15 (50%)	18 (20%)	0.002
Baseline Serum Thyroglobulin (ng/mL)	13.1 (9.9–51.8)	23.5 (9.8–66.2)	0.32
Baseline Thyroglobulin Antibody (ng/mL)	45.5 (17–118)	11 (2–42)	0.0003
Mortality from Thyroid cancer	2 (6.7%)	5 (5.6%)	0.99
Thyroid cancer recurrence rates	12 (40%)	28 (31.1%)	0.38
Elapsed duration since initial postoperative thyroid marker conduction (years)	5.7 (4–7.1)	7.1 (5–8.2)	0.28
Elapsed duration since Thyroid cancer diagnosis (years)	5.5 (4–7)	7 (5–8)	0.22

#### 3.2.2 Impact on survival

**Figure 1:**
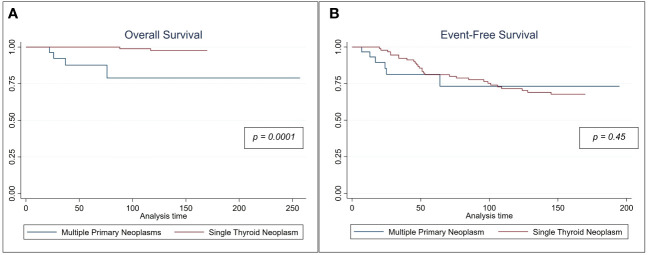
Kaplan Meir Survival curves comparing **(A)** the cancer specific overall survival and **(B)** event-free survival for patient with multiple neoplasms vs those who had single thyroid malignancy.

With regards to cancer-specific OS, patients with MPN had worse OS than those with TC alone. At a median follow-up of 135 months, MPN patients were found to have a poorer 5-year survival than patients with TC alone (*p* < 0.01). However, the 5-year EFS was not significantly different between these two subcohorts (**Figure 1**). Both univariate and multivariable Cox hazard models (**Table 5**), suggest that the presence of MPN can have a negative effect on OS (*p*≤ 0.02).

**Table 5:** Univariate and multivariate survival analyses for overall survival.

Univariate Analysis			
Variable	Hazard Ratio	95% Confidence Interval	*p*-value
Age at diagnosis	1.1	0.9–1.2	0.78
Number of Neoplasms	2.5	1.1–5.1	0.001
Obesity	0.4	0.08–1.6	0.16
Smoking	1.7	0.4–8.3	0.52
Family History	0.1	0.1–65.3	0.37
Abnormal Serum Thyroglobulin	1.1	0.2–8.1	0.89
Abnormal Thyroglobulin Antibody	1.2	0.2–10.9	0.91
Multivariate Analysis			
Variable	Hazard Ratio	95% Confidence Interval	*p*-value
Age At diagnosis	0.98	0.97–1.1	0.56
Number of Neoplasms	22.6	1.6–324.8	0.02
Obesity	0.09	0.003–2.8	0.18
Smoking	2.7	0.3–26.9	0.42
Family History	0.2	0.1–3.2	0.92
Abnormal Serum Thyroglobulin	2.5	0.2–38.7	0.49
Abnormal Thyroglobulin Antibody	1.9	0.1–30.3	0.63

## 4 Discussion

Our research underscores an important finding regarding patients with MPN, revealing a notably lower survival rate. According to our study, MPN patients were diagnosed at an older age, displayed an elevated body mass index, and presented with increased thyroglobulin antibody levels, along with a more pronounced familial history of cancer.

The global rise in TC incidence is a widely recognized trend, largely attributed to the progress in clinical procedures, biochemical knowledge, and imaging technologies (38). The evolution of medical guidelines and practices, such as more thorough evaluations of the thyroid gland, has resulted in higher rates of TC detection. Progress in understanding the biochemical aspects of TC, including the identification of new biomarkers and genetic mutations linked to the disease, has also been a significant factor (39). Additionally, the widespread use of advanced ultrasound technology has facilitated the identification of small thyroid nodules, including thyroid microcarcinomas (38). In our MPN subcohort, imaging played a crucial role in the initial detection and evaluation of TC, underscoring the importance of ongoing clinical monitoring and multidisciplinary care for both cancer survivors and sufferers. However, advancements in therapeutic and diagnostic techniques have drawbacks, as previous research has linked the occurrence of secondary cancer and MPN to exposure to radiation (40, 41). While radiation therapy is effective in treating various types of cancer, it is also associated with an increased risk of developing additional primary malignancies. The carcinogenic impact of radiation on the thyroid gland primarily stems from the DNA damage caused by ionizing radiation, leading to genetic mutations and chromosomal abnormalities that initiate cancer formation. Additionally, exposure to radiation significantly heightens the likelihood of developing thyroid cancer, with the risk increasing in proportion to the dosage received, even at lower levels (40).

With regards to TC treatment, effective management strategies involve a multidisciplinary approach that incorporates surgical, oncological, medical, and radiological assessments (42). Surgical intervention has been found to reduce mortality and event rates in cases of operable TC, even when dealing with microcarcinomas (43). Furthermore, research has indicated that radioactive iodine therapy can significantly enhance survival outcomes for individuals with differentiated TC (44–47). For example, a large study involving 96,557 patients demonstrated that the use of radioactive iodine was linked to improved disease-specific survival in this population (48). Furthermore, radioactive iodine therapy has been shown to improve rates of recurrence-free survival, by eliminating residual thyroid tissue and microscopic disease (49). This treatment is particularly advantageous for patients with certain risk factors, such as extrathyroidal extension or lymph node involvement, as it reduces the likelihood of cancer recurrence (50).

MPN are infrequent, exhibiting a greater occurrence in older patients (51). The occurrence of MPN is predominantly noted in cancer survivors as well as in cancer subtypes that are associated with favorable outcomes. Therefore, the rising prevalence of TC and the favorable survival outcomes have resulted in a higher occurrence of MPN (52). However, MPN in survivors of TC may contribute significantly to mortality rates. This emphasizes the need for vigilant surveillance of MPN occurrence to enhance prognosis. In our study population consisting of 563 individuals, we identified 30 patients who developed MPNs, indicating a prevalence of approximately 5% at our institution, which closely aligns with prior findings from Taiwan (26). Lin et al. outlined that TC patients with MPN exhibit a lower female-to-male ratio, an older mean age, and more advanced TNM stages compared to those with only TC (26). These patients also receive higher doses of therapeutic radioactive iodine and have a higher total mortality rate (26). Notably, hematological malignancies and renal cell carcinoma are prevalent as secondary cancers in these patients, despite not being among the top ten common cancers in the general population of Taiwan, indicating a distinct pattern of secondary malignancies in thyroid cancer patients (26).

The possibility of being diagnosed with another neoplasm after surviving a first one is 14% higher for people who have previously had cancer compared to the general population (53). This percentage is anticipated to increase due to the growing number of cancer survivors. Consequently, there has been a rise in the occurrence of MPN among individuals who have overcome cancer, accounting for almost one-fifth of all newly diagnosed malignancies in recent studies (54).

With regards to MPN incidence, there is a notable variation in the reported incidence shared in current literature ranging from 1–37% (55). The variation observed in these findings can be attributed to the different methodologies used for calculations and the diverse ethnic populations studied. In the United States, the prevalence of primaries has been reported in percentages over time. For example, it was found to be 3.5% in New Mexico in 1977, 5.3% according to a study conducted in 1968, and finally 8% based on data from 1975–2001 (56–58). Similarly, in Western Europe, the occurrence of MPN varied across studies; it was recorded as 0.7% in a hospital registry in Scotland in 1972, 6.3% based on European cancer registries from 1995 to 1999, and reached up to 11.7% according to an autopsy series from Sweden in 1969 (59–61). The literature also shows variation in the numbers and types of multiple primaries in relation to the total number of cancer patients. For example, the incidence of three or more primaries ranged from 0.2% in a cancer center in Jordan (2006–2011) to 2.0% in a cancer center in France (1980–2009) (62–64). Similarly, metachronous primaries accounted for 3% in a recent study from the United States (2005–2012) which is equivalent to our obtained results (55).

Currently, there is a lack of a definitive risk factor that has been confirmed for the development of MPN. However, various factors have been previously investigated. It has been hypothesized that the presence of MPN in cancer survivors may be influenced by increased vigilance or can be a result of previous treatments, and could also be linked to mutual risk factors such as environmental factors, and genetic predisposition (60). In addition, ethnic background is a significant determinant of susceptibility to certain diseases (55). Various societies exhibit varying rates of occurrence for specific types of neoplasms (14). For instance, the Japanese population experiences a notable prevalence of gastric cancer, while having a relatively low incidence of prostate cancer (65). Conversely, the Jordanian population demonstrates a considerably higher occurrence of prostate and lung malignancies (66).

There is a strong correlation between obesity and the development of cancer in the general population, specifically in relation to breast cancer, genitourinary cancers, and gastrointestinal cancers (67). Therefore, obesity has been found to significantly increase the likelihood of developing MPN. This connection was observed in the current study, which found that patients with MPN were more likely to be obese than those with a single form of malignancy. In addition, the higher levels of baseline thyroglobulin antibodies in patients with MPN can point to the possibility of autoimmune thyroid disease. A recent cohort study by Chen et al. concluded that patients with autoimmune thyroid disease are more susceptible to developing various cancers, including breast, colorectal, and head and neck cancers (68). They also found that elderly patients with autoimmune thyroid disease can have an increased risk of developing thyroid and colorectal cancer compared with the general population (68). Therefore, clinicians must be aware of such associations in the clinical oncology care.

Family history is a significant risk factor for the development of MPN. There is a notable presence of familial clustering across almost all cancer sites, and this clustering is primarily observed in close relationships within families spanning multiple generations (69). The majority of hereditary cancers display an excess of multiple primary tumors and an early onset of the disease (67). This clustering phenomenon may be attributed to abnormal genes or gene variants. Various cancer predisposition genes, including BRCA1, BRCA2, and p16/CDKN2A, have already been identified in high-risk pedigrees (67, 69). Additionally, individuals with hereditary nonpolyposis colorectal cancer who have MPN often exhibit a higher frequency of mismatch repair genes, such as hMSH2 and hMLH1 (70). Moreover, cribriform variants of PTC were identified as being classically linked with familial adenomatous polyposis (71).

Breast cancer and TC are among the most prevalent forms of cancer in women (72). In our study, we found a higher incidence of breast cancer in MPN patients. Breast cancer was found to be the most common secondary cancer in patients with TC, occurring either simultaneously, prior to, or after the TC diagnosis. This bidirectional relationship between thyroid and breast cancer has been previously suggested in a retrospective study, which also found an increased risk of developing a MPN in patients with a history of breast or TC (73). Various factors, such as iodine transport, obesity, sex hormones, thyroid hormones, and cancer treatment, have been potentially implicated in the development of both types of cancer (73, 74). Lei et al. explored the prognosis of breast cancer patients who subsequently developed metachronous TC using information obtained from the Surveillance, Epidemiology, and End Results database (75). The study aimed to compare the prognosis of breast cancer patients who had a second primary thyroid cancer with those who did not, analyzing a wide range of clinicopathological and treatment characteristics, and evaluating the impact of second metachronous TC on breast cancer survival. The population studied included breast cancer patients diagnosed between 2000 and 2014, with a subset of these patients developing a second primary TC within this timeframe. The study found that the impact of other malignancies on TC prognosis (and vice versa) can vary depending on the type and timing of the secondary cancer, as well as the specific characteristics of the cancer itself. This study contributes significantly to understanding the complex interplay between primary and secondary malignancies, highlighting the importance of considering the type, timing, and specific characteristics of secondary cancers in the prognosis of the primary cancer (75).

The presentation of hematologic malignancy with a second primary neoplasm is most commonly observed during the follow-up period (76). Breast, lung, and TC are the most frequently occurring secondary tumors (76). Typically, secondary TC in these patients occurs at a later time. It has been established that radiotherapy administered at the early stages of lymphoma is linked to an increased risk of developing secondary TC (77). Another study demonstrated that prior radiotherapy of the head, neck, and upper diaphragm may also contribute to the development of TC (78). In lymphoma patients, a recent study found that diffuse large B-cell lymphoma was the most commonly observed hematologic malignancy alongside TC in MPN patients (76). Similar to our findings, PTC was the most coinciding malignancy discovered incidentally (76).

Based on our analysis, it was found that patients with MPN exhibited a lower survival rate. This is more in line with previous research findings, as MPN had shown a detrimental and statistically significant impact on the survival of patients with TC (52). Liou et al. found that the overall mortality rate in TC patients with MPN could be 4.4 times higher compared to those without MPN (79). Similarly, Lang et al. reported a considerable mortality rate of 43.5% in TC patients with nonsynchronous MPN (80).

This study faces several limitations, chiefly due to its retrospective nature, a limited number of patients, and being conducted at a single institution. Moreover, despite attempts to mitigate confounding variables through propensity score matching and the exclusion of genetic conditions, the retrospective approach may have compromised accurate final selection due to the lack of exhaustive details on genetic testing, thyroid marker assays, and laboratory analyses, among other factors. Despite this, the present study is the first to address this topic using propensity score-matching among MPN patients of Middle-Eastern ethnicity.

## 5 Conclusion

The onset of MPN can significantly influence the survival outcome of TC patients. The diagnosis of thyroid carcinoma at an older age, accompanied by elevated initial thyroglobulin antibody levels and a notable familial predisposition, may raise concerns about the potential occurrence of synchronous or metachronous neoplastic manifestations. Further analysis with larger patient cohorts is necessary to confirm the results of this study.

## Data availability statement

The raw data supporting the conclusions of this article will be made available by the authors, without undue reservation.

## Ethics statement

The studies involving humans were approved by Institutional Review Board of King Hussein Cancer Center (KHCC) in Amman, Jordan (Registration number: 22 KHCC 131, 11 August 2022). The studies were conducted in accordance with the local legislation and institutional requirements. The participants provided their written informed consent to participate in this study. Written informed consent was obtained from the individual(s) for the publication of any potentially identifiable images or data included in this article.

## Author contributions

A-AI: Conceptualization, Data curation, Formal Analysis, Funding acquisition, Investigation, Methodology, Project administration, Resources, Software, Supervision, Validation, Visualization, Writing – original draft, Writing – review & editing. ASA: Conceptualization, Data curation, Formal Analysis, Investigation, Methodology, Software, Writing – original draft, Writing – review & editing. DA-A: Conceptualization, Data curation, Formal Analysis, Investigation, Methodology, Project administration, Writing – original draft, Writing – review & editing. EL: Conceptualization, Data curation, Formal Analysis, Investigation, Methodology, Project administration, Software, Supervision, Writing – original draft, Writing – review & editing. AA-O: Conceptualization, Writing – original draft, Writing – review & editing. MA-M: Conceptualization, Investigation, Writing – original draft, Writing – review & editing. YY: Conceptualization, Data curation, Formal Analysis, Writing – original draft, Writing – review & editing. NA-H: Writing – original draft, Writing – review & editing. IM: Writing – original draft, Writing – review & editing. SS: Writing – review & editing, Methodology, Writing – original draft. GS: Conceptualization, Formal Analysis, Methodology, Validation, Writing – original draft, Writing – review & editing.

## References

[1] Pettersson B Adami HO Wilander E Coleman MP. Trends in thyroid cancer incidence in Sweden, 1958–1981, by histopathologic type. Int J Cancer. (1991) 48:28–33. doi: 10.1002/ijc.2910480106 2019455

[2] dos Santos Silva I Swerdlow A. Thyroid cancer epidemiology in England and Wales: time trends and geographical distribution. Br J Cancer. (1993) 67:330–40. doi: 10.1038/bjc.1993.61 PMC1968194 8431362

[3] Haselkorn T Bernstein L Preston-Martin S Cozen W Mack WJ. Descriptive epidemiology of thyroid cancer in Los Angeles County, 1972–1995. Cancer Causes Control. (2000) 11:163–70. doi: 10.1023/A:1008932123830 10710201

[4] Burgess JR. Temporal trends for thyroid carcinoma in Australia: an increasing incidence of papillary thyroid carcinoma (1982–1997). Thyroid. (2002) 12:141–9. doi: 10.1089/105072502753522374 11916283

[5] Reynolds RM Weir J Stockton DL Brewster DH Sandeep TC Strachan MW. Changing trends in incidence and mortality of thyroid cancer in Scotland. Clin Endocrinol (Oxf). (2005) 62:156–62. doi: 10.1111/j.1365-2265.2004.02187.x 15670190

[6] Davies L Welch HG. Increasing incidence of thyroid cancer in the United States, 1973–2002. JAMA. (2006) 295:2164–7. doi: 10.1001/jama.295.18.2164 16684987

[7] Hayat MJ Howlader N Reichman ME Edwards BK. Cancer statistics, trends, and multiple primary cancer analyses from the Surveillance, Epidemiology, and End Results (SEER) Program. oncologist. (2007) 12:20–37. doi: 10.1634/theoncologist.12-1-20 17227898

[8] Verkooijen HM Fioretta G Pache J-C Franceschi S Raymond L Schubert H. Diagnostic changes as a reason for the increase in papillary thyroid cancer incidence in Geneva, Switzerland. Cancer Causes Control. (2003) 14:13–7. doi: 10.1023/A:1022593923603 12708720

[9] Vecchia CL Ron E Franceschi S Maso LD Mark SD Chatenoud L. A pooled analysis of case-control studies of thyroid cancer. III. Oral contraceptives, menopausal replacement therapy and other female hormones. Cancer Causes Control. (1999) 10:157–66. doi: 10.1023/A:1008832513932 10231164

[10] Hemminki K Li X. Gender effects in familial cancer. Int J Cancer. (2002) 102:184–7. doi: 10.1002/ijc.10676 12385016

[11] Sherman SI Perrier N Clayman GL. Thyroid Cancer. Springer. (2012), 295–310. doi: 10.1007/978-1-4614-5197-6_26

[12] Sant M Aareleid T Berrino F Lasota MB Carli P Faivre J. EUROCARE-3: survival of cancer patients diagnosed 1990–94—results and commentary. Ann Oncol. (2003) 14:v61–v118. doi: 10.1093/annonc/mdg754 14684501

[13] Gilliland FD Hunt WC Morris DM Key CR. Prognostic factors for thyroid carcinoma: A population-based study of 15,698 cases from the Surveillance, Epidemiology and End Results (SEER) Program 1973–1991. Cancer: Interdiscip Int J Am Cancer Society. (1997) 79:564–73. doi: 10.1002/(ISSN)1097-0142 9028369

[14] Al-Ibraheem A Al-Rasheed U Mashhadani N Abdlkadir AS Al-Adhami DA Ruzzeh S. Long-term survival analysis and prognostic factors of arabic patients with differentiated thyroid carcinoma: A 20-year observational study at the King Hussein Cancer Center (KHCC) involving 528 patients. Cancers (Basel). (2023) 15:4102. doi: 10.3390/cancers15164102 37627130 PMC10452119

[15] Baloch ZW Asa SL Barletta JA Ghossein RA Juhlin CC Jung CK. Overview of the 2022 WHO classification of thyroid neoplasms. Endocr Pathol. (2022) 33:27–63. doi: 10.1007/s12022-022-09707-3 35288841

[16] Pstrąg N Ziemnicka K Bluyssen H Wesoły J. Thyroid cancers of follicular origin in a genomic light: in-depth overview of common and unique molecular marker candidates. Mol Cancer. (2018) 17:1–17. doi: 10.1186/s12943-018-0866-1 30089490 PMC6081953

[17] Rossi ED Pantanowitz L Hornick JL. A worldwide journey of thyroid cancer incidence centered on tumor histology. Lancet Diabetes Endocrinol. (2021) 9:193–4. doi: 10.1016/S2213-8587(21)00049-8 33662332

[18] Li Y Che W Yu Z Zheng S Xie S Chen C. The incidence trend of papillary thyroid carcinoma in the United States during 2003–2017. Cancer Control. (2022) 29:10732748221135447. doi: 10.1177/10732748221135447 36256588 PMC9583193

[19] Ulisse S Baldini E Lauro A Pironi D Tripodi D Lori E. Papillary thyroid cancer prognosis: An evolving field. Cancers (Basel). (2021) 13:5567. doi: 10.3390/cancers13215567 34771729 PMC8582937

[20] Badulescu C Piciu D Apostu D Badan M Piciu A. Follicular thyroid carcinoma-clinical and diagnostic findings in a 20-year follow up study. Acta Endocrinologica (Bucharest). (2020) 50:170. doi: 10.4183/aeb.2020.170 PMC7535902 33029233

[21] Nagaiah G Hossain A Mooney CJ Parmentier J Remick SC. Anaplastic thyroid cancer: a review of epidemiology, pathogenesis, and treatment. J Oncol. (2011) 2011: 1–13. doi: 10.1155/2011/542358 PMC3136148 21772843

[22] Al-Ibraheem A Alyasjeen SF Abdlkadir AS Sheikha AA. [68Ga] Ga-DOTA-FAPI-04 PET/CT depicts metastases from medullary thyroid cancer that [68Ga] Ga-DOTATOC PET/CT missed. Eur J Nucl Med Mol Imaging. (2023), 1–2. doi: 10.1007/s00259-023-06348-4 37490080

[23] Sandeep TC Strachan MW Reynolds RM Brewster DH Scélo G Pukkala E. Second primary cancers in thyroid cancer patients: a multinational record linkage study. J Clin Endocrinol Metab. (2006) 91:1819–25. doi: 10.1210/jc.2005-2009 16478820

[24] Rubino C De Vathaire F Dottorini M Hall P Schvartz C Couette J. Second primary Malignancies in thyroid cancer patients. Br J Cancer. (2003) 89:1638–44. doi: 10.1038/sj.bjc.6601319 PMC2394426 14583762

[25] Ronckers CM McCarron P Ron E. Thyroid cancer and multiple primary tumors in the SEER cancer registries 1. Int J Cancer. (2005) 117:281–8. doi: 10.1002/ijc.21064 15880372

[26] Lin J Lin K Chao T Hseuh C Tsang N Huang B. Clinical presentations of thyroid cancer patients with multiple primary cancers. J Endocrinological Invest. (2011) 34:824–30. doi: 10.3275/7747 21613811

[27] Adjadj É Rubino C Shamsaldim A Lê MG Schlumberger M de Vathaire F. The risk of multiple primary breast and thyroid carcinomas: Role of the radiation dose. Cancer: Interdiscip Int J Am Cancer Society. (2003) 98:1309–17. doi: 10.1002/cncr.11626 12973856

[28] Oemuer O Oezcan Z Yazici B Akgün A Oral A Oezkilic H. Multiple primary tumors in differentiated thyroid carcinoma and relationship to thyroid cancer outcome. Endocr J. (2008) 55:365–72. doi: 10.1507/endocrj.K07E-058 18277003

[29] Tucker M Boice J Jr Hoffman D. Second cancer following cutaneous melanoma and cancers of the brain, thyroid, connective tissue, bone, and eye in Connecticut, 1935–82. Natl Cancer Institute Monograph. (1985) 68:161–89. 4088297

[30] Osterlind A Olsen JH Lynge E Ewertz M. Second cancer following cutaneous melanoma and cancers of the brain, thyroid, connective tissue, bone, and eye in Denmark, 1943–80. Natl Cancer Institute Monograph. (1985) 68:361–88. 4088310

[31] Teppo L Pukkala E Saxén E. Multiple cancer—an epidemiologic exercise in Finland. J Natl Cancer Inst. (1985) 75:207–17. 3860679

[32] Levi F Randimbison L Te V Rolland-Portal I Franceschi S La Vecchia C. Multiple primary cancers in the Vaud Cancer registry, Switzerland, 1974–89. Br J Cancer. (1993) 67:391–5. doi: 10.1038/bjc.1993.72 PMC1968177 8431373

[33] Amin MB Edge SB Greene FL Byrd DR Brookland RK Washington MK. AJCC cancer staging manual Vol. 1024. New Jersey, United States: Springer (2017).

[34] Li S Ren C Gong Y Ye F Tang Y Xu J. The role of thyroglobulin in preoperative and postoperative evaluation of patients with differentiated thyroid cancer. Front Endocrinol (Lausanne). (2022) 13:872527. doi: 10.3389/fendo.2022.872527 35721746 PMC9200986

[35] Warren S Gates O. A survey of the literature and statistical study. Am J Cancer. (1932) 16:1358–4142.

[36] Coyte A Morrison DS McLoone P. Second primary cancer risk-the impact of applying different definitions of multiple primaries: results from a retrospective population-based cancer registry study. BMC Cancer. (2014) 14:1–11. doi: 10.1186/1471-2407-14-272 24742063 PMC4005906

[37] Nosé V. Familial thyroid cancer: a review. Mod Pathol. (2011) 24:S19–33. doi: 10.1038/modpathol.2010.147 21455198

[38] Lim H Devesa SS Sosa JA Check D Kitahara CM. Trends in thyroid cancer incidence and mortality in the United States, 1974–2013. JAMA. (2017) 317:1338–48. doi: 10.1001/jama.2017.2719 PMC8216772 28362912

[39] Grogan RH Mitmaker EJ Clark OH. The evolution of biomarkers in thyroid cancer—From mass screening to a personalized biosignature. Cancers (Basel). (2010) 2:885–912. doi: 10.3390/cancers2020885 24281099 PMC3835110

[40] Iglesias ML Schmidt A Ghuzlan AA Lacroix L Vathaire F Chevillard S. Radiation exposure and thyroid cancer: a review. Arch Endocrinol Metab. (2017) 61:180–7. doi: 10.1590/2359-3997000000257 PMC10118869 28225863

[41] Brown AP Chen J Hitchcock YJ Szabo A Shrieve DC Tward JD. The Risk of Second Primary Malignancies up to Three Decades after the Treatment of Differentiated Thyroid Cancer. J Clin Endocrinol Metab. (2008) 93:504–15. doi: 10.1210/jc.2007-1154 18029468

[42] Mallick UK Harmer C. Practical management of thyroid cancer: A multidisciplinary approach. Berlin, Germany: Springer (2018). doi: 10.1007/978-3-319-91725-2

[43] Bi J Zhang H. A meta-analysis of total thyroidectomy and lobectomy outcomes in papillary thyroid microcarcinoma. Medicine. (2023) 102. doi: 10.1097/MD.0000000000036647 PMC10727648 38115346

[44] Li C Wu Q Sun S. Radioactive iodine therapy in patients with thyroid carcinoma with distant metastases: a SEER-based study. Cancer Control. (2020) 27:1073274820914661. doi: 10.1177/1073274820914661 32292051 PMC7160783

[45] Tang J Kong D Cui Q Wang K Zhang D Liao X. The role of radioactive iodine therapy in papillary thyroid cancer: an observational study based on SEER. Onco Targets Ther. (2018) 11:3551–60. doi: 10.2147/OTT PMC6016280 29950860

[46] Chai J Zhang R Zheng W Zhang G Jia Q Tan J. Predictive value of clinical and pathological characteristics for metastatic radioactive iodine-refractory differentiated thyroid carcinoma: a 16-year retrospective study. Front Endocrinol (Lausanne). (2022) 13:930180. doi: 10.3389/fendo.2022.930180 35846335 PMC9281388

[47] Al-Ibraheem A Al-Shammaa M Abdlkadir AS Istatieh F Al-Rasheed U Pascual T. Survival trends in pediatric differentiated thyroid cancer: A middle eastern perspective. Life. (2024) 14:158. doi: 10.3390/life14010158 38276287 PMC10820815

[48] Alshwayyat SA Al-Akhras A Ababneh OE Alshwayyat TA Ghazou A. The effect of radioactive iodine (RAI) on disease-specific survival in differentiated thyroid cancer (DTC): A study of 96,557 patients. J Clin Oncol. (2023) 41:e18095–5. doi: 10.1200/JCO.2023.41.16_suppl.e18095

[49] Iizuka Y Katagiri T Ogura K Inoue M Nakashima R Nakamura K. Recurrence-free survival and prognosis after adjuvant therapy with radioactive iodine-131 in patients with differentiated thyroid carcinoma. Sci Rep. (2023) 13:10795. doi: 10.1038/s41598-023-37899-z 37402838 PMC10319734

[50] Liu X Fan Y Liu Y He X Zheng X Tan J. The impact of radioactive iodine treatment on survival among papillary thyroid cancer patients according to the 7th and 8th editions of the AJCC/TNM staging system: a SEER-based study. Updates Surg. (2020) 72:871–84. doi: 10.1007/s13304-020-00773-y 32342347

[51] Demandante CGN Troyer DA Miles TP. Multiple primary Malignant neoplasms: case report and a comprehensive review of the literature. Am J Clin Oncol. (2003) 26:79–83. doi: 10.1097/00000421-200302000-00015 12576929

[52] Wu ST Chi SY Wang PW Chen YN Yang YT Chen WC. Analysis of overall survival in differentiated thyroid cancer patients with double primary Malignancy. Kaohsiung J Med Sci. (2021) 37:63–71. doi: 10.1002/kjm2.12286 32841516 PMC11896261

[53] Liu L de Vries E Louwman M Aben K Janssen-Heijnen M Brink M. Prevalence of multiple Malignancies in the Netherlands in 2007. Int J Cancer. (2011) 128:1659–67. doi: 10.1002/ijc.25480 20503267

[54] Travis LB Rabkin CS Brown LM Allan JM Alter BP Ambrosone CB. Cancer survivorship—genetic susceptibility and second primary cancers: research strategies and recommendations. J Natl Cancer Inst. (2006) 98:15–25. doi: 10.1093/jnci/djj001 16391368

[55] Amer MH. Multiple neoplasms, single primaries, and patient survival. Cancer Manag Res. (2014) 6:119–34. doi: 10.2147/CMAR PMC3949559 24623992

[56] Mariotto AB Rowland JH Ries LA Scoppa S Feuer EJ. Multiple cancer prevalence: a growing challenge in long-term survivorship. Cancer Epidemiol Biomarkers Prev. (2007) 16:566–71. doi: 10.1158/1055-9965.EPI-06-0782 17372253

[57] Bordin GM Key CR McQuade CE Kutvirt DM Hughes WB Brylinski DA. Multiple primary cancers. Relative risk in New Mexico’s triethnic population. Cancer. (1977) 40:1793–800. doi: 10.1002/(ISSN)1097-0142 907983

[58] Hajdu SI Hajdu EO. Multiple primary Malignant tumors. J Am Geriatr Soc. (1968) 16:16–26. doi: 10.1111/j.1532-5415.1968.tb03965.x 5634468

[59] Haddow A Boyd J Graham A. Multiple primary neoplasms in the Western Hospital Region, Scotland: a survey based on cancer registration data. Scott Med J. (1972) 17:143–52. doi: 10.1177/003693307201700404 5023391

[60] Rosso S Terracini L Ricceri F Zanetti R. Multiple primary tumors: incidence estimation in the presence of competing risks. Population Health metrics. (2009) 7:1–10. doi: 10.1186/1478-7954-7-5 19338658 PMC2682788

[61] Berge T Cederqvist L Schönebeck J. Multiple primary Malignant tumors: an autopsy study of a circumscribed population. Acta Pathologica Microbiologica Scandinavica. (1969) 76:171–83. doi: 10.1111/j.1699-0463.1969.tb03248.x 5373627

[62] Salem A Abu-Hijlih R Abdelrahman F Turfa R Amarin R Farah N. Multiple primary Malignancies: analysis of 23 patients with at least three tumors. J Gastrointest Cancer. (2012) 43:437–43. doi: 10.1007/s12029-011-9296-7 21706155

[63] Watanabe S Kodama T Shimosato Y Arimoto H Sugimura T Suemasu K. Multiple primary cancers in 5,456 autopsy cases in the National Cancer Center of Japan. J Natl Cancer Inst. (1984) 72:1021–7. 6585580

[64] Pagès P-B Mordant P Grand B Badia A Foucault C Dujon A. History of multiple previous Malignancies should not be a contraindication to the surgical resection of lung cancer. Ann Thorac Surgery. (2013) 95:1000–5. doi: 10.1016/j.athoracsur.2012.11.072 23375734

[65] Tomoda H Taketomi A Baba H Kohnoe S Seo Y Saito T. Multiple primary colorectal and gastric carcinoma in Japan. Oncol Rep. (1998) 5:147–56. doi: 10.3892/or 9458311

[66] Khader YS Sharkas GF Arkoub KH Alfaqih MA Nimri OF Khader AM. The epidemiology and trend of cancer in Jordan, 2000–2013. J Cancer Epidemiol. (2018) 2018. doi: 10.1155/2018/2937067 PMC6207872 30416523

[67] Soerjomataram I Coebergh JW. Epidemiology of multiple primary cancers. Cancer Epidemiol. (2009) 85–105. doi: 10.1007/978-1-59745-416-2_5 19109776

[68] Chen Y-K Lin C Cheng FT Sung F Kao C. Cancer risk in patients with Hashimoto’s thyroiditis: a nationwide cohort study. Br J Cancer. (2013) 109:2496–501. doi: 10.1038/bjc.2013.597 PMC3817335 24084773

[69] Albright F Teerlink C Werner TL Cannon-Albright LA. Significant evidence for a heritable contribution to cancer predisposition: a review of cancer familiarity by site. BMC Cancer. (2012) 12:1–7. doi: 10.1186/1471-2407-12-138 22471249 PMC3350420

[70] Artac M Bozcuk H Ozdogan M Demiral AN Sarper A Samur M. Different clinical features of primary and secondary tumors in patients with multiple Malignancies. Tumori J. (2005) 91:317–20. doi: 10.1177/030089160509100406 16277096

[71] Cameselle-Teijeiro JM Peteiro-González D Caneiro-Gómez J Sánchez-Ares M Abdulkader I Eloy C. Cribriform-morular variant of thyroid carcinoma: a neoplasm with distinctive phenotype associated with the activation of the WNT/β-catenin pathway. Mod Pathol. (2018) 31:1168–79. doi: 10.1038/s41379-018-0070-2 29785019

[72] Nielsen SM White MG Hong S Aschebrook-Kilfoy B Kaplan EL Angelos P. The breast–thyroid cancer link: a systematic review and meta-analysis. Cancer Epidemiol Biomarkers Prev. (2016) 25:231–8. doi: 10.1158/1055-9965.EPI-15-0833 PMC4770576 26908594

[73] An JH Hwangbo Y Ahn HY Keam B Lee KE Han W. A possible association between thyroid cancer and breast cancer. Thyroid. (2015) 25:1330–8. doi: 10.1089/thy.2014.0561 26442580

[74] Dong L Lu J Zhao B Wang W Zhao Y. Review of the possible association between thyroid and breast carcinoma. World J Surg Oncol. (2018) 16:1–7. doi: 10.1186/s12957-018-1436-0 29976206 PMC6034293

[75] Lei K He X Yu L Ni C Chen H Guan D. Breast cancer prognosis is better in patients who develop subsequent metachronous thyroid cancer. PloS One. (2019) 14:e0215948. doi: 10.1371/journal.pone.0215948 31042767 PMC6493754

[76] Li Q Zhu F Xiao Y Liu T Liu X Zhang L. Synchronous double primary lymphoma and thyroid cancer: A single-institution retrospective study. Medicine. (2021) 100. doi: 10.1097/MD.0000000000027061 PMC8483851 34596109

[77] Schaapveld M Aleman BM van Eggermond AM Janus CP Krol AD van der Maazen RW. Second cancer risk up to 40 years after treatment for Hodgkin’s lymphoma. N Engl J Med. (2015) 373:2499–511. doi: 10.1056/NEJMoa1505949 26699166

[78] Sigurdson AJ Ronckers CM Mertens AC Stovall M Smith SA Liu Y. Primary thyroid cancer after a first tumor in childhood (the Childhood Cancer Survivor Study): a nested case-control study. Lancet. (2005) 365:2014–23. doi: 10.1016/S0140-6736(05)66695-0 15950715

[79] Liou M-J Tsang N-M Hsueh C Chao T-C Lin J-D. Therapeutic outcome of second primary Malignancies in patients with well-differentiated thyroid cancer. Int J Endocrinol. (2016) 2016. doi: 10.1155/2016/9570171 PMC4828550 27118971

[80] Lang BH-H Wong KP. Risk factors for nonsynchronous second primary Malignancy and related death in patients with differentiated thyroid carcinoma. Ann Surg Oncol. (2011) 18:3559–65. doi: 10.1245/s10434-011-1777-1 PMC3222830 21573833

